# Real-time PCR and immunohistochemistry detection of *Wolbachia* in adult *Dirofilaria immitis* from dogs treated with doxycycline and ivermectin

**DOI:** 10.1186/s13071-025-06720-3

**Published:** 2025-02-26

**Authors:** Yi Chu, Kaori Sakamoto, Christopher C. Evans, Michael T. Dzimianski, Crystal Fricks, Abdelmoneim Mansour, Utami DiCosty, Scott McCall, John W. McCall, C. Thomas Nelson, Andrew R. Moorhead

**Affiliations:** 1 Department of Pathology, College of Veterinary Medicine, University of Georgia, Athens, GA USA; 2 Department of Population Health and Pathobiology, College of Veterinary Medicine, North Carolina State University, Raleigh, NC USA; 3 Department of Infectious Diseases, College of Veterinary Medicine, University of Georgia, Athens, GA USA; 4 TRS Labs Inc., P.O. Box 5112, Athens, GA USA; 5 VCA Animal Medical Center of NE Alabama, Anniston, AL USA; 6 Department of Clinical Sciences, College of Veterinary Medicine, North Carolina State University, Raleigh, NC, USA

**Keywords:** Doxycycline, *Dirofilaria immitis*, *Wolbachia*, Immunohistochemistry, Quantitative PCR

## Abstract

**Background:**

*Wolbachia* is present in all life stages of *Dirofilaria immitis*. *Wolbachia* surface protein (WSP) can be highly immunogenic and induce acute inflammatory reactions in the host upon worm death. To avoid the abrupt release of *Wolbachia* and its antigens from deceased parasites, the American Heartworm Society (AHS) has recommended using doxycycline (DOXY) and having a 1-month wait period between the DOXY treatment and the adulticidal process for *Wolbachia* elimination. Studies have shown that the 28 day, 10 mg/kg twice daily (BID) administration of DOXY can effectively clear *Wolbachia* in the bloodstream of the host. The 1-month wait period is hypothesized to allow for further reduction of *Wolbachia*. However, the levels of *Wolbachia* in adult parasites after the DOXY treatment remain unknown.

**Methods:**

Forty-five purposely bred dogs were intravenously transplanted with 20 *Dirofilaria immitis* adults, consisting of 12 females and 8 males. The dogs were divided into nine groups of five dogs each. Two groups each received 5, 7.5, or 10 mg/kg DOXY BID orally for 28 days, and ivermectin (IVM) monthly (6 µg/kg.) Three groups remained untreated as controls. Study animals were necropsied on day 0, day 30, and day 60, following the start of treatment. Adult worms were collected at necropsy and preserved for analysis. Quantitative polymerase chain reaction (qPCR) and immunohistochemistry for WSP were performed on worms collected at each time point. The data were analyzed using a linear mixed model (LMM). Multiple comparisons were adjusted using Tukey’s test.

**Results:**

The qPCR results showed that all treatment doses significantly reduced *Wolbachia* levels compared with the control groups at 30 and 60 days. The intradose comparison indicated a significant decrease on day 60 compared with day 30. No significant differences were found between different doses on the two examination dates. Immunohistochemistry indicated the markedly reduced presence of *Wolbachia* in treatment groups.

**Conclusions:**

All DOXY dosages can be considered effective in reducing *Wolbachia* on both tested dates (30 and 60 days). On the basis of the further reduction of *Wolbachia* levels in adult *D. immitis*, the 1-month rest period in the AHS heartworm treatment guidelines is beneficial. *Wolbachia* can still be detected on day 60 in all dosage groups.

**Graphical Abstract:**

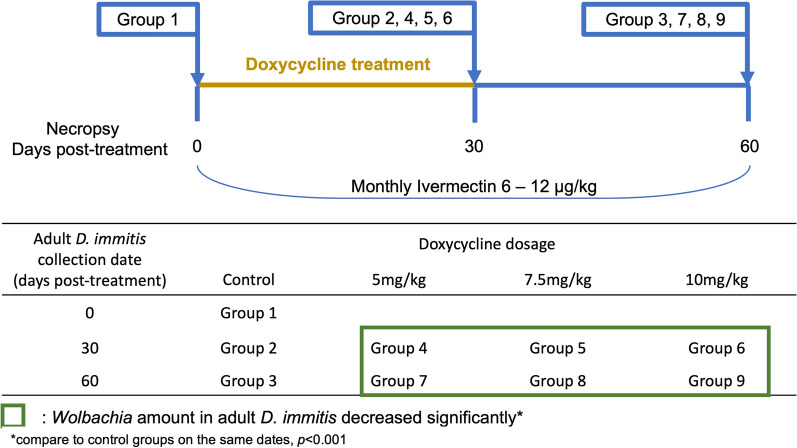

**Supplementary Information:**

The online version contains supplementary material available at 10.1186/s13071-025-06720-3.

## Background

Heartworm disease, caused by *Dirofilaria immitis*, is a long-existing and continuing threat to the well-being of canine species worldwide. *Dirofilaria immitis* requires an intermediate host (mosquito) and definitive hosts to complete its life cycle. *Dirofilaria immitis* adults can live in the pulmonary artery of the host for up to 7 years and produce microfilariae (mf) if both adult males and females are present. To treat the parasites, the American Heartworm Society (AHS) recommends three injections of melarsomine dihydrochloride (MEL), with 1 month between the first and second doses, and 24 h between the second and third doses, to kill the adult parasites. Prior to the adulticidal treatment, the AHS recommends the use of a macrocyclic lactone along with doxycycline (DOXY) as a supplementary treatment [1].

Doxycycline is a tetracycline-class antibiotic that can treat a wide range of infections caused by bacteria, including *Mycoplasma* and *Rickettsia* species [2]. Doxycycline is also used as an adjunct therapy in the treatment of elephantiasis, dirofilariasis, and onchocerciasis, by targeting the endosymbiont *Wolbachia* [3]. *Wolbachia* is hypothesized to perform biosynthetic activities that were found to be absent in the nematode genome, including heme utilization, lipid synthesis, and enzyme metabolism [4–7]. The *Wolbachia* genus belongs to the Alphaproteobacteria class in the order Rickettsiales. This bacterium is found in many filarial nematodes in the family *Onchocercidae*, including *Brugia malayi*, *B. pahangi*, *Onchocerca volvulus*, and *Dirofilaria immitis*, in all life stages. In human filariasis and onchocerciasis, *Wolbachia* surface protein (WSP), in a manner similar to lipopolysaccharide, induces a pro-inflammatory innate immune reaction by activating host monocytes, macrophages, dendritic cells, and neutrophils [8, 9]. When using diethylcarbamazine and/or ivermectin (IVM) without DOXY, post-treatment reactions, including fever, tachycardia, headaches, and lymph node enlargement, can happen after treatment of *B. malayi* and onchocerciasis infection [10, 11]. Though the roles of *Wolbachia* in filarial nematodes are not fully understood, multiple studies have shown that the lack of *Wolbachia* impacts the viability and fertility of the parasite in different developmental stages [6, 12–14]. The elimination of *Wolbachia* can reduce the inflammatory immune reactions to bacteria released from the parasite, as well as prevent the potential pathological changes that the inflammation may induce.

Compared with other anthelmintic treatments alone, the addition of DOXY showed increased efficiency in mf elimination and parasite killing [15, 16]. However, the optimal dosage of DOXY in heartworm treatment remains unclear. Members of our group have previously investigated the elimination of *D. immitis* mf and adults, as well as lung pathology of the hosts, using different dosages of IVM and DOXY either alone or together [17, 18]. In this previous work, doxycycline was given at 10 mg/kg once per day orally, starting 6 weeks after adult *D.immitis* transplantation, with or without IVM. The groups that received MEL treatment started at week 24 and followed the AHS protocol. All dogs were euthanized at week 36. Both DOXY and IVM showed significant efficacy in mf reduction, including the group that received both treatments. Meanwhile, the copy number of the *Wolbachia* *ftsZ* gene in adult worms was significantly decreased in DOXY and DOXY + IVM treatment groups. *Wolbachia* surface protein (WSP) immunohistochemistry indicated a reduction in *Wolbachia* in DOXY and DOXY + IVM groups, but not in the IVM-only group [18]. Histopathology of the MEL and DOXY + IVM + MEL groups suggested a reduction in lung lesions in animals that received DOXY + IVM [19]. A clinical study [20] showed a total clearance of circulating *Wolbachia* DNA in antigen- and mf-positive dogs (*n* = 17) after 10 mg/kg, twice per day, 30-day DOXY administration with 2.5% moxidectin.

*Dirofilaria immitis* infection can induce cardiovascular and pulmonary lesions in multiple ways. Kramer et al. assessed lung pathology in *D. immitis*-experimentally infected dogs treated with MEL, with or without prior DOXY treatment [21]. Doxycycline was given 20 mg/kg orally, once per day, for 4 weeks. Melarsomine injection started 8 weeks after the completion of DOXY treatment, as per AHS recommendations. Dogs treated with MEL alone formed typical lung lesions associated with thromboembolism, and severe alveolar wall thickening and hepatization, while dogs that received DOXY pretreatment showed decreased severity of the lesions, especially with regard to perivascular inflammation.

A clinical study by Nelson et al. [22] looked at the rates of respiratory complications and heartworm disease-related deaths in dogs naturally infected with heartworm that received DOXY 10 mg/kg BID prior to MEL injection (*n* = 47) versus no DOXY before MEL (*n* = 47). All dogs received MEL 30 days after the completion of DOXY. They found fewer respiratory complications and heartworm disease-related deaths in the DOXY treated group compared with the no DOXY group (6.52% versus 19.14%; 0% versus 4.25%, respectively).

The present study aimed to investigate *Wolbachia* levels in adult *D. immitis* after DOXY treatment at different dosages, as well as before and after the 1-month wait period (also referred to as the “rest period”) at the end of the DOXY regimen. We evaluated the necessity of the 1-month wait period on the basis of changes in *Wolbachia* levels in adult *D. immitis* at the beginning and end of the 1-month “rest period.”

## Methods

### Parasites

Donor animals were infected with approximately 250 *D. immitis* third-stage larvae (GA-3 isolate) via subcutaneous injection into the flank. The adult worms were collected at 6.5 months post-infection.

### Study animals

**Fig. 1:**
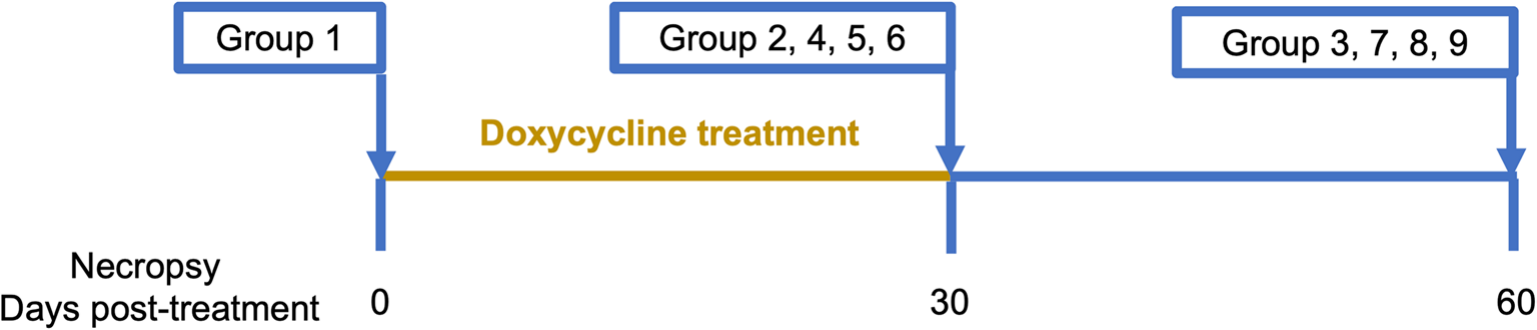
Experimental timeline

All animals used in this study were housed in a specific pathogen-free facility for the duration of the study. Forty-five young purpose-bred adult dogs were intravenously transplanted with 20 adults (6.5 months old) *D. immitis* (GA-3 isolate, 12 females and 8 males) each, and were randomly divided into nine groups of five dogs each. All animals were housed and treated as described previously, with the approval of the Institutional Animal Care and Use Committee (IACUC) [23]. Briefly, the dogs and transplanted worms were allowed to rest for 75 days before DOXY and IVM treatment was started. Groups 1, 2, and 3 served as non-treated controls. Groups 4–9 received different DOXY regimens and monthly IVM (Table 1). Groups 4 and 7, groups 5 and 8, and groups 6 and 9 received BID DOXY at 5 mg/kg, 7.5 mg/kg, or 10 mg/kg, respectively. All treatment groups received monthly ivermectin at the preventive dose of 6–12 µg/kg. On day 0 post-treatment, control group 1 was euthanized and necropsied. On day 30 post-treatment, control group 2 and treatment groups 4, 5, and 6 were euthanized and necropsied. On day 60, control group 3 and treatment groups 7, 8, and 9 were euthanized and necropsied (Fig. 1). Adult worms were collected, sexed, labeled, and stored at necropsy in 70% ethanol for DNA analysis or 4% paraformaldehyde (PFA) for anti-WSP immunohistochemistry (IHC) staining.

**Table 1:** Experimental treatment design for all groups

Necropsy (days post-treatment)	Control	DOXY 5 mg/kg	DOXY 7.5 mg/kg	DOXY 10 mg/kg
		IVM 6 µg/kg monthly (all treatment groups)		
0	Group 1			
30	Group 2	Group 4	Group 5	Group 6
60	Group 3	Group 7	Group 8	Group 9

### DNA isolation and quantitative real-time PCR

Three adult female and male *D. immitis* from each dog were used for DNA isolation. One adult female and one adult male were randomly picked from the extra worms used for intravenous transplantation and served as standard controls for all female and male samples, respectively. Each single worm was soaked overnight in 50 mL phosphate-buffered saline (PBS) before liquid nitrogen homogenization. The worms were broken into powder, aliquoted per the suggestion of the DNeasy Blood & Tissue kit (QIAGEN, Valencia, CA), and processed according to the kit protocol. Blood samples of groups 2 to 9 were collected on the day of necropsy with ethylenediaminetetraacetic acid (EDTA) tubes. The whole blood samples were kept at −80 °C till DNA isolation. The samples were thawed at room temperature and processed per the DNeasy Blood & Tissue kit (QIAGEN, Valencia, CA) protocol. Isolated DNA was stored at −20 °C until further analysis.

Relative quantitative PCR (qPCR) was performed to determine the ratio of *Wolbachia* *ftsZ* DNA to *D. immitis* 18S DNA to assess the change in *Wolbachia* levels in adult worms and in whole blood. Primers were chosen based on previous research [24]. The *Wolbachia* amplicon (GenBank: AJ495000) included forward (5′-GCT GGT GCC TTA CCT GAT ATT-3′) and reverse (5′-CCA CCC ATT CCT GCT GTT AT-3′) primers to amplify a 110-bp fragment. The *D. immitis* amplicon (GenBank: AF036638) included forward (5′-TGA GAA ACG GCT ACC ACA TC-3′) and reverse (5′-GAT AAC CGG CCT CAT AGA GAA C-3′) primers that amplified a 112-bp fragment. The assay used SsoAdvanced™ Universal SYBR^®^ Green Supermix (Bio-Rad, Hercules, CA) with Bio-Rad CXF96 Touch Real-Time PCR Detection System. Raw data were collected with CFX Maestro Software (Bio-Rad).

### Immunohistochemistry staining

Two female and two male, adult *D. immitis* from each group were randomly selected for immunohistochemistry. The adult worms were fixed in 4% paraformaldehyde and embedded in paraffin for sectioning. The worms were kept static in 10% agarose and trimmed to expose the cross-section before embedding. Briefly, the slides were deparaffinized, rehydrated, blocked with peroxidase suppressor and blocking buffer, incubated with primary and secondary antibodies, and stained using a horseradish peroxidase (HRP) staining technique with AEC (3-amino-9-ethylcarbazole) substrate, according to the recommendations of the manufacturers (BD PharmingenTM AEC Substrate Kit; BD Biosciences, catalog # 551015) and as described in the supplementary material protocol (Additional file 1: Text S1). The primary antibody was an anti-WSP monoclonal antibody (BEI Resources, NIAID, NIH. NR-51684) with the dilution of 1:250; mouse IgG2a kappa isotype control (Invitrogen. Catalog no. 14–4724) served as negative control. The goat anti-mouse IgG (H + L) secondary antibody, HRP (Invitrogen. REF 32430) was used at a dilution of 1:50. ProlongTM Gold anti-fade Mountant (Invitrogen, REF P10144) was used, and all stained slides were photographed within 48 h to avoid the fading of color.

### Microfilariae counting

Blood samples were collected on days −2, 7, 13, 20, 27, 34, 41, 48, 55, and 60 of the study. At each blood collection, microfilariae were counted as described in previous publications [17, 18]. Briefly, 20 µL blood was used to prepare slides, which were stained with Giemsa stain and examined under the microscope. The 1 mL blood samples were mixed with 2% buffered formalin and kept for the modified Knott test.

### Data analysis

The qPCR results were collected as Ct values. All Ct values were processed by CFX Manager software (Bio-Rad, Hercules, CA, USA) and 2^−ΔΔCt^ (fold change) was calculated using the Livak method [25]. The readouts for the *Wolbachia* *fstZ* gene for each sample were normalized, first to the internal control (*D. immitis* 18 s rRNA gene), then to the standard control (one adult male or female *D. immitis* from the same batch of transplantation). The fold change of *Wolbachia* is presented in the descriptive data. The fold change data were log-transformed to match the assumptions of normality and homoscedasticity of model residuals to apply to a linear mixed model. All analyses were performed using SAS 9.4 (Cary, NC, USA). Multiple comparisons were adjusted by using Tukey’s test. The graphs were made with GraphPad Prism version 10.0.0 (Boston, MA, USA).

## Results

### Real-time PCR detection of *Wolbachia* in adult *D. immitis*

**Fig. 2:**
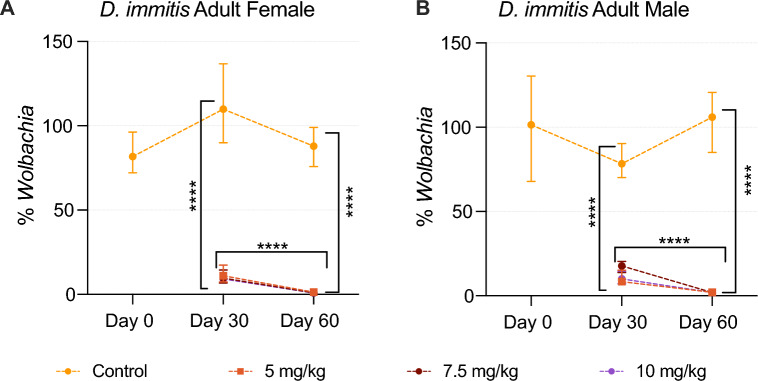
Relative *Wolbachia* levels in *D. immitis* female (**A**) and male (**B**) adults for each group at each time point showed as percentage-fold change. Horizontal asterisks indicate significant differences between time points within each dosage of treatment groups (intra-dose comparison between days, *p* < 0.0001). Vertical asterisks indicate significant differences between treatment (5 mg/kg, 7.5 mg/kg, and 10 mg/kg) and control groups at a single time point (all *p* < 0.0001)

Within *D. immitis* female adults recovered at necropsy, the *Wolbachia* levels in all treatment groups were significantly decreased from the control groups (control versus 5 mg/kg; control versus 7.5 mg/kg; control versus 10 mg/kg) on day 30 (all *p* < 0.0001, *df* = 36; *t*_*df*_ = 5.85, *t*_*df*_ = 7.43, and *t*_*df*_ = 7.61, respectively) and day 60 (all *p* < 0.0001, *df* = 36; *t*_*df*_ = 12.22, *t*_*df*_ = 14.47, *t*_*df*_ = 14.11, respectively) on the basis of statistical analysis performed on log e transformed fold-change data. No significant differences were found between different dosage groups (5 mg/kg versus 7.5 mg/kg; 5 mg/kg versus 10 mg/kg; 7.5 mg/kg versus 10 mg/kg) on day 30 (*df* = 36; *p* = 0.40, *t*_*df*_ = 1.58; *p* = 0.31, *t*_*df*_ = 1.76; and *p* = 1.00, *t*_*df*_ = 0.18, respectively) or day 60 (*df* = 36, *p* = 0.13, *t*_*df*_ = 2.25; *p* = 0.25, *t*_*df*_ = 1.89; *p* = 0.98, *t*_*df*_ = −0.36, respectively). Between days 30 and 60, all dosage groups (5 mg/kg, 7.5 mg/kg, and 10 mg/kg) showed a significant decrease in *Wolbachia* levels within each dosage (all *p* < 0.0001; *df* = 36; *t*_*df*_ = 6.83, *t*_*df*_ = 7.50, and *t*_*df*_ = 6.96, respectively) (Fig. 2A).

The *Wolbachia* levels in *D. immitis* male adults presented a similar decline over time. All treatment groups displayed a significant decrease in *Wolbachia* levels compared with control groups (control versus 5 mg/kg; control versus 7.5 mg/kg; control versus 10 mg/kg) on day 30 (all *p* < 0.0001, *df* = 36; *t*_*df*_ = 8.51, *t*_*df*_ = 6.16, and *t*_*df*_ = 7.67, respectively) and day 60 (all *p* < 0.0001, *df* = 36; *t*_*df*_ = 14.67, *t*_*df*_ = 15.04, and *t*_*df*_ = 15.77, respectively). Among all dosage groups (5 mg/kg versus 7.5 mg/kg; 5 mg/kg versus 10 mg/kg; 7.5 mg/kg versus 10 mg/kg), no significant differences were found on day 30 (*df* = 36; *p* = 0.11, *t*_*df*_ = −2.35; *p* = 0.84, *t*_*df*_ = −0.84; and *p* = 0.44, *t*_*df*_ = 1.51, respectively) or day 60 (*df* = 36; *p* = 0.98, *t*_*df*_ = 0.37; *p* = 0.69, *t*_*df*_ = 1.10; and *p* = 0.89, *t*_*df*_ = 0.73, respectively) post-treatment. The intradose comparisons between days revealed a significant decrease in *Wolbachia* levels on day 60 compared with day 30 within all treatment groups (5 mg/kg, 7.5 mg/kg, 10 mg/kg; all *p* < 0.0001; *df* = 36; *t*_*df*_ = 5.37, *t*_*df*_ = 8.10, and *t*_*df*_ = 7.31, respectively) (Fig. 2B).

Tables 2 and 3 summarize the *Wolbachia* levels in female and male adults in percentages of control standard. To quantify the changes in *Wolbachia* levels, we used the Livak method to calculate the relative *Wolbachia* levels. We defined the *Wolbachia* levels in control standards as 100%, and all data are shown in percentages of the control standards. The interquartile range (IQR) indicates the range of *Wolbachia* levels that include 50% of the population given certain conditions (DOXY dosages, examination date). The amount of *Wolbachia* in adult *D. immitis* significantly decreased, both before (all *p* < 0.0001) and after the 1-month wait period (all *p* < 0.0001). On day 30 post-treatment, the median *Wolbachia* levels in adult females were reduced to 11.1% (5 mg/kg DOXY), 9.6% (7.5 mg/kg DOXY), and 9.2% (10 mg/kg DOXY) of the female control standard. On day 60 post-treatment, the median *Wolbachia* levels in adult females further decreased to 1.3% (5 mg/kg DOXY), 0.9% (7.5 mg/kg DOXY), and 0.8% (10 mg/kg DOXY) of the control standard. The median *Wolbachia* levels in adult males showed a similar trend. On days 30 and 60 post-infection, the median *Wolbachia* levels were at 8.4% and 2.1% (5 mg/kg DOXY), 17.7% and 1.9% (7.5 mg/kg DOXY), and 10.1% and 1.9% (10 mg/kg DOXY) of the male control standard, respectively.

**Table 2:** Real-time PCR data summary of relative *Wolbachia* levels in adult female *D. immitis*. Percentages were calculated using the ΔΔCt method

Percentage of *Wolbachia* in adult female *D. immitis*				
Days post-treatment	Group	DOXY	% median	% interquartile range (IQR)
0	1	Control	81.8	(72.1, 96.3)
30	2	Control	109.9	(89.9, 136.8)
	4	5 mg/kg	11.1	(7.4, 17.3)
	5	7.5 mg/kg	9.6	(6.8, 14.4)
	6	10 mg/kg	9.2	(7.1, 11.3)
60	3	Control	88	(75.8, 99.1)
	7	5 mg/kg	1.3	(1.0, 2.1)
	8	7.5 mg/kg	0.9	(0.9, 1.0)
	9	10 mg/kg	0.8	(0.5, 1.6)

**Table 3:** Real-time PCR data summary of relative *Wolbachia* levels in adult male *D. immitis*. Percentages were calculated using the ΔΔCt method

Percentage of *Wolbachia* in adult male *D. immitis*				
Days post-treatment	Group	DOXY	% Median	% Interquartile range (IQR)
0	1	Control	101.4	(67.9, 130.4)
30	2	Control	78.4	(70.1, 90.4)
	4	5 mg/kg	8.4	(6.2, 15.2)
	5	7.5 mg/kg	17.7	(13.8, 20.4)
	6	10 mg/kg	10.1	(7.1, 14.6)
60	3	Control	106.0	(85, 120.7)
	7	5 mg/kg	2.1	(1.4, 2.8)
	8	7.5 mg/kg	1.9	(1.3, 2.8)
	9	10 mg/kg	1.9	(1.0, 2.1)

### Real-time PCR detection of *Wolbachia* DNA in whole blood

The percentage of *Wolbachia* DNA detected in whole blood samples is presented in Table 4. Samples were defined as negative in *Wolbachia* DNA when the Ct values of the real-time PCR were equal to or higher than 35, or no DNA was amplified. All animals in control groups 2 and 3 tested positive on days 30 and 60, respectively. On day 30, three out of five animals in group 4 (DOXY 5 mg/kg, BID) were negative. Animals in other treatment groups had 100% negative testing results for *Wolbachia* DNA.

**Table 4:** Percentage of *Wolbachia* DNA detection in whole blood

Group	DOXY	Necropsy day	
		30 (groups 2, 4, 5, 6)	60 (groups 3, 7, 8, 9)
2, 3	Control	100	100
4, 7	5 mg/kg	40	0
5, 8	7.5 mg/kg	0	0
6, 9	10 mg/kg	0	0

Quantitative PCR was performed on DNA isolated from whole blood collected on the day of necropsy. Primers were designed to detect *D. immitis* 18S and *Wolbachia ftsZ* genes with SYBR green dye. Samples were categorized as negative for the presence of *Wolbachia* DNA if Ct values were ≥ 35 or no DNA was amplified

### Immunohistochemistry

**Fig. 3:**
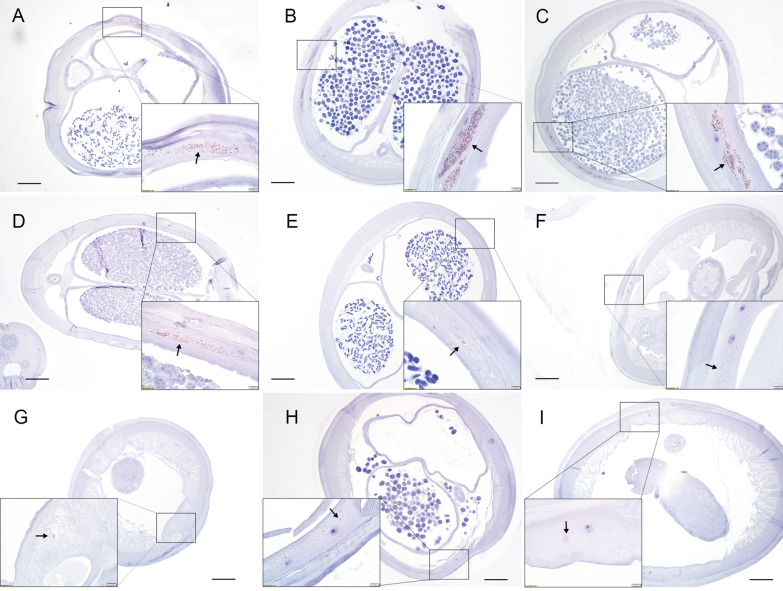
Detection of *Wolbachia* in adult female *D. immitis* by immunohistochemistry with anti-WSP antibody. The rectangular box indicates 10× magnification. Scale bar 100 µM. **A**–**C** Cross-section from groups 1, 2, and 3 (control groups necropsied on 0, 30, 60 days post-treatment), respectively. Dense *Wolbachia* clusters (arrows) in the lateral cord. **D**–**F** Cross-sections from groups 4, 5, and 6 (DOXY 5 mg/kg, 7.5 mg/kg, and 10 mg/kg, necropsied 30 days post-treatment), respectively. Scattered *Wolbachia* clusters (arrows) in the lateral cord. **G**–**I** Cross-sections from groups 7, 8, and 9 (DOXY 5 mg//kg, 7.5 mg/kg, and 10 mg/kg, necropsied 60 days post-treatment), respectively. Single *Wolbachia* (arrows in **G**, **H**) and deformed *Wolbachia* (arrows in **I**) in the lateral cord

**Fig. 4:**
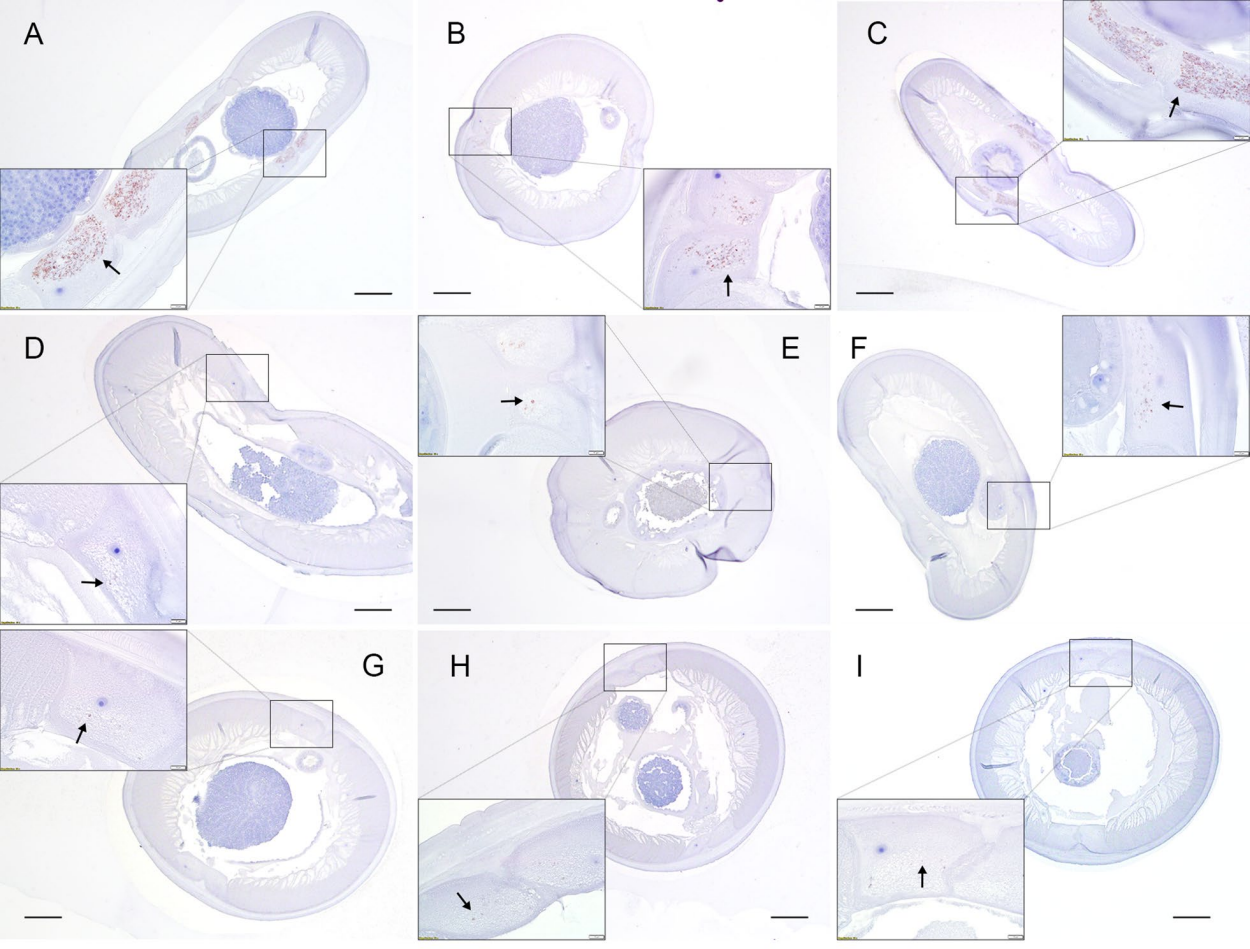
Detection of *Wolbachia* in adult male *D. immitis* by immunohistochemistry with anti-WSP antibody. The rectangular box indicates 10× magnification. Scale bar 100 µM. **A**–**C** Cross-section from groups 1, 2, and 3 (control groups necropsied on 0, 30, and 60 days post-treatment), respectively. Dense *Wolbachia* clusters (arrows) in the lateral cord. **D**–**F** Cross-sections from groups 4, 5, and 6 (DOXY 5 mg/kg, 7.5 mg/kg, and 10 mg/kg, necropsied 30 days post-treatment), respectively. Scattered *Wolbachia* (arrows) in lateral cord. **G**–**I** Cross-sections from groups 7, 8, and 9 (DOXY 5 mg/kg, 7.5 mg/kg, and 10 mg/kg, necropsied 60 days post-treatment), respectively. Single *Wolbachia* (arrows) in the lateral cord

*Dirofilaria immitis* female and male adults were sectioned and stained with anti-WSP antibody. *Wolbachia* clusters were present in the lateral cord of female and male adults from the control groups across the dates examined (Figs. 3 and 4, respectively). All samples were immunopositive for WSP despite treatment with DOXY and the time at which the sample was collected. Dense, robust clusters of *Wolbachia* were present in the control female and male adults (Fig. 3A–C; Fig. 4A–C). Fewer *Wolbachia* were observed in groups 4, 5, and 6 (all dosages at day 30) in both female and male adults (Fig. 3D–F; Fig. 4D–F). Single, scattered, and deformed *Wolbachia* were observed in groups 7, 8, and 9 (day 60, all dosages; Fig. 3G–I; Fig. 4G–I). The reduction of positive staining of WSP aligned with the real-time PCR results.

### Microfilariae count

The mean mf counts of each group are presented in Supplementary Fig. S1. The number of mf of each animal is shown in Additional file 2: Table S1. All animals were mf positive prior to the DOXY and IVM treatment (2 days after the start point of DOXY treatment), ranging from 8300–27600 mf/mL. No clear trend of changes in mean mf count were observed in groups 1, 2, 4, 5, and 6. Groups 7, 8, and 9 showed gradually decreased mf count, beginning approximately 34 days post-treatment. Two days prior to necropsy, all animals from groups 1, 2, 4, 5, and 6 had mf. At necropsy, all animals in groups 3 and 9 had mf. Two of the five animals in group 7 were amicrofilaremic. Two of the five animals in group 8 had 0 and 100 mf/mL, respectively.

## Discussion

The recently updated AHS heartworm treatment protocol indicated the use of 10 mg/kg DOXY for 28 days before adulticidal treatment to both reduce the pathology resulting from dead and dying worms, and prevent the development of L3s in subsequent hosts [1]. Our study focused on the *Wolbachia* levels in adult worms after treatment with IVM and different dosages of DOXY. In this study, we examined the efficacy of multiple DOXY dosages in reducing *Wolbachia* immediately after completion of the course of DOXY, and 1 month later. Our results suggest that the relative *Wolbachia* numbers in adult *D. immitis* females and males decrease significantly after DOXY and IVM treatment among all dosage groups (Fig. 2; Tables 2, 3). The percentage of positive *Wolbachia* DNA detection in blood decreased as the treatment was processed (Table 4). The observation of mf count was performed in a relatively short period compared with other studies (60 days versus 36 weeks) [17, 18], but a trend of the decrease can be seen in groups 7, 8, and 9 (Additional file 2: Supplementary Table S1; Additional file 3: Supplementary Fig. S1), beginning at approximately day 34 after the start of DOXY and IVM treatment.

The 1-month wait period after DOXY treatment, and before the first melarsomine injection, was hypothesized to clear the *Wolbachia* and WSP that may persist in the worms [1]. Our data show that *Wolbachia* levels were significantly decreased after the 1-month wait period for each dosage group (all *p* < 0.0001). This finding aligns with the hypothesis and suggests that conserving the 1-month wait period would be beneficial in reducing complications caused by *Wolbachia*.

Although a significant decrease in *Wolbachia* was observed among all treatment groups, none showed complete clearance of *Wolbachia*, as indicated by the qPCR and immunohistochemistry staining results. *Wolbachia* surface protein persisted in the highest dosage group, even after the 1-month wait period (10 mg/kg DOXY, Fig. 3I). No statistical analysis can be performed on the staining results; however, a visible decrease in WSP can be observed in all treatment groups on days 30 and 60 post-treatment. It should be noted, however, that owing to the size and thickness of the cuticles of adult *D. immitis*, the worms were stiff post fixation and difficult to bend or place at a certain angle or position for histology. To obtain cross-sections of the worms, we stabilized the samples in 10% agarose before trimming and embedding in paraffin. We could not control the way worms were held in the 10% agarose. Thus, the cross-section obtained from each worm was random. The *Wolbachia* in *D. immitis* adults are not evenly distributed, as described in a previous publication [26], which may cause bias when interpreting the IHC data. We observed a general decrease in positive staining (data not shown) and captured representative sections from each group (Figs. 3, 4). If cross-sections at the exact same location for each worm could have been obtained, we may have been able to perform statistical analysis according to the surface area of positive staining. We did not, however, find any positive staining in any of the study animal tissue sections, including those that contained dead worms, from the control groups and treatment groups (data not shown). This may be due to the dead worm cuticles remaining intact and, thus, preventing *Wolbachia* from entering the hosts’ tissue. These negative staining results align with the histopathology scores from a recent study by Moorhead et al. [23], which used the same group of experimental animals and conditions, indicating no significant differences in lung and pulmonary artery histopathology scores between the control and the treatment groups.

In Moorhead et al. [23], we compared the mean worm weight change between groups. For all dose groups, this was significantly increased from the start of the DOXY treatment (day 0) to the completion of the DOXY treatment (day 30), and then significantly decreased after the rest period (day 60). Our current study showed that the *Wolbachia* levels in adult worms were significantly decreased in treatment groups 30 days post-treatment, which could have impacted the reproductive system of *D. immitis*. However, we cannot determine whether the decrease in *Wolbachia* correlated with the changes in mean worm weight. The decrease in mean worm weight 60 days post-treatment, compared with 30 days, may be due to a negative impact of *Wolbachia* elimination on mf production and the general health of adult *D. immitis*. The increase in mean weight changes shown in 30 day post-treatment groups may be due to the natural growth of the adult *D. immitis*. Although the *Wolbachia* levels decreased significantly in the 30-day post-treatment groups, our study (Supplementary Table S1; Supplementary Fig. S1) and other studies have shown that the elimination of circulating mf, when treated with DOXY with or without IVM, takes longer than 30 days [17, 18, 24], which may suggest that the embryos or other reproduction-related structures in *D. immitis* remain functional at 30 days post-treatment.

This is the first study that has been able to link the amount of *Wolbachia* in adult *D. immitis* to different DOXY dosages. The data we presented could aid in decision-making for practitioners. Our results suggest that the 1-month wait period may be beneficial, as the *Wolbachia* levels further reduced, compared with 30 days post-treatment, with statistical significance. As shown in Tables 2 and 3, the *Wolbachia* levels decreased to 8.4–17.7% of standard control in all treatment groups at the completion of treatment, then further decreased to 0.8–2.1% after the 1-month rest period. However, we did not perform the AHS-recommended MEL injections to kill the adult worms at the end of the study, as the intact adult worms were needed to quantitate the *Wolbachia*. Thus, we could not correlate the *Wolbachia* levels in adult worms to potential complications or pathologies induced by WSP or other *Wolbachia* metabolites following MEL treatment. A study by Kramer et al. [21] evaluated the severity of lesions in experimentally infected *D. immitis* dogs treated with DOXY alone [20 mg/kg once daily (SID) for 4 weeks] or DOXY and IVM (20 mg/kg DOXY SID for 4 weeks, 6 µg/kg IVM monthly) before administration of MEL (8 weeks after the completion of DOXY) versus MEL alone. Their study showed that the treatment with DOXY, which could lead to the depletion of *Wolbachia*, showed reduced pathology lesion scores following MEL injection. Owing to the limitations stated above, they did not perform assays to quantify the *Wolbachia* levels in the parasite at the time of MEL injection. They inferred that the reduction in populations of *Wolbachia* could reduce the potential lesions. However, whether the further decrease of *Wolbachia* after the rest period, as we found in our current study (for example, 17.7% to 2.1%; Table 3; groups 4 and 7), will significantly impact the complications and lesion formation after MEL injection, requires additional study using our experimental design.

We found no statistically significant differences in *Wolbachia* levels between the dosages of DOXY on days 30 and 60, and between these days. Though this study was not designed to test for equality of the efficacy of DOXY against *Wolbachia*, this finding may suggest the use of reduced DOXY dosages in situations where the ideal dose of 10 mg/kg cannot be used. A study has shown that DOXY given at less than 10 mg/kg, with the monthly Heartgard^®^ Plus (ivermectin/pyrantel), may lead to an increased time for mf clearance from the peripheral blood of the host [24]. As shown in our study, the detection of *Wolbachia* DNA in whole blood aligns with the previous findings [24]; that on day 28 of DOXY administration, 10 mg/kg DOXY had a 100% reduction in *Wolbachia* DNA detection, while 5 mg/kg DOXY showed less efficacy.

We should note that while DOXY is targeted at *Wolbachia* in heartworm and other filarial nematode treatments, this antibiotic also shows efficacy in anti-inflammatory and immunomodulatory activities, which may aid in the reduction of pathology [2]. Another limitation of this study is that we transplanted the adult *D. immitis* instead of natural infection, in order to assess treatments for the same worm burden (20 worms total). In clinical practice, other factors, such as the age and health condition of the patients, worm burden, and the compliance of the pet owners may also impact the efficacy of the treatment. Our study can potentially inform the decision-making process in the choice of DOXY dosages as well as the necessity of the 1-month wait period.

## Conclusions

Our study showed that all dosages of DOXY effectively decreased *Wolbachia* levels in *D. immitis* at both time points (days 30 and 60 following the start of treatment). A lower dosage of DOXY may be considered if the patient does not tolerate DOXY well at the recommended dosage (10 mg/kg, BID). The *Wolbachia* levels in *D. immitis* adults decreased further after the 1-month wait period but were still present. The 1-month rest period in the AHS heartworm treatment guidelines is potentially beneficial and should be maintained at present.

## Supplementary Information

Additional file 1. Text S1: anti-WSP *D. immitis* AEC staining.

Additional file 2. Table S1: Microfilariae count in blood

Additional file 3. Supplementary Fig. S1. Mean microfilaria counts for groups. Fig. S1. Mean microfilaria counts for groups necropsied on days 0 and 30 (S1-A) and on day 60 (S1-B), beginning 2 days prior to the start of DOXY and IVM treatment. Groups 1–3: control groups with no treatment; groups 4, 7: 5 mg/kg DOXY; groups 5, 8: 7.5 mg/kg DOXY; groups 6, 9: 10 mg/kg DOXY. Doxycycline was given twice daily for 28 days. All treatment groups were given monthly IVM.

## Data Availability

Data are provided within the manuscript. The uncurated data that support the findings of this study are available from the corresponding author upon reasonable request.
