# Supplementary material for: Real-time PCR and immunohistochemistry detection of Wolbachia in adult Dirofilaria immitis from dogs treated with doxycycline and ivermectin
Source: Parasit Vectors. 2025 Feb 26;18:78. doi: 10.1186/s13071-025-06720-3 (PMC11866827; doi:10.1186/s13071-025-06720-3)
Supplement: Supplementary file 1 — Additional file 1. Text S1: anti-WSP D. immitis AEC staining. [file 13071_2025_6720_MOESM1_ESM.docx]

Anti-WSP immunohistochemistry AEC staining protocol

Section 1: Reagents and materials

Section 2: Preparation

Section 3: Procedure

Section 4: Notes

Section 1

| P-10 pipette  P-50 pipette  P-200 pipette  Pipette tips  Coplin jars | Timer  Marker  Pap pen  Mayer’s Hematoxylin (EMS 26853-04)  Aqueous mounting media |
| --- | --- |
| Monoclonal Anti-Wolbachia Surface Protein (Immunoglobulin G, Mouse) BEI, Cata# NR-51684 | |
| Pierce Peroxidase IHC Detection Kit  (ThermoFisher Cata# 36000) including:  Peroxidase Suppressor  Global blocking buffer  BD Pharmingen™ AEC substrate set (Cata# 551015) including:  AEC substrate  AEC Chromogen | Goat anti-Mouse IgG (H+L) Secondary Antibody, HRP. Thermo Fisher Cata# 32430  Mouse IgG2a kappa Isotype Control (eBM2a). Thermo Fisher Cata# 14-4724-81  Prepared Wash buffer* |
| Paper towel  Foil wrap  Kim wipe | Distilled water  Beaker |
| Fume hood** | |
| 100%, 95%, 70% EtOH in Coplin jars  Xylene in Coplin jar X 2 | |
| Slide baker | |

*Store wash buffer in 4^o^C refrigerator once prepared. Prepare fresh blocking buffer for each experiment. Wash buffer may store up to 2 weeks.

**Use Xylene in the fume hood only.

Section 2

- Reagent Preparation

| 95% Ethanol | Absolute ethanol 190mL + 10mL distill water |
| --- | --- |
| 70% Ethanol | Absolute ethanol 140mL + 60mL distill water |
| Wash buffer | 1 Pack of BupH Tris Buffered Saline (from kit) + 500mL distill water + 2.5mL 10% Tween-20. Store at 4^o^C. |
| AEC working solution* | One drop (20uL) of AEC Chromogen with 1mL of AEC substrate. Vortex to mix. |

*Make working solution **NO MORE than 15 minutes prior to use**.

Bring the Wash buffer to room temperature before starting the experiment.

Section 3 *****Once paraffin is removed, make sure the section stays moist.**

- **Paraffin Removal and Rehydration**

1. Bake slides 60^o^C for 40 minutes.
2. All incubations in paraffin removal steps are performed in Coplin jars.
3. Incubate slides for 5 minutes in xylene; repeat once by changing the Coplin jar. Tap off excess liquid on a paper towel.
4. Incubate slides for 3 minutes in 100% ethanol; repeat once. Tap off excess liquid.
5. Incubate slides for 3 minutes in 95% ethanol; repeat once. Tap off excess liquid.
6. Incubate slides for 5 minutes in 70% ethanol. Tap off excess liquid.
7. Incubate slides for 1 minute in 0.1M PBS.
8. Carefully wipe around the section with Kim wipe and circle the sample with pap pen. Make sure that the pap pen is dried before the next step. Pap pen may take 15 – 20 seconds to dry.

- **Endogenous Peroxidase Inhibition**

1. Put the slide horizontally in a humidity chamber. Add peroxidase suppressor enough to cover the sections. 200uL liquid should be enough to cover one slide. Adjust the volume if needed. Make sure to cover the samples on the slides. Incubate for 30 minutes at room temperature.
2. Tap off the peroxidase suppressor, wash slide for 3 minutes with wash buffer in Coplin jar, and repeat once.

- **Staining**

1. Put the slides horizontally in a humidity chamber. Block for 10 minutes with blocking buffer at room temperature. Cover the humidity chamber with foil paper.
2. Dilute primary antibody and isotype control at 1: 250 with blocking buffer. Make the volume enough to cover the samples on the slides.
3. Incubate with primary antibody for 60 minutes in a humidity chamber at room temperature. This step can be done at 37^o^C for 30 minutes.
4. Wash slides for 3 minutes with wash buffer. Repeat once.
5. Dilute HRP-conjugated secondary antibody at 1:50 with blocking buffer.
6. Incubate with secondary antibody for 30 minutes in a humidity chamber at room temperature and cover with foil wrap.
7. Wash slides for 3 minutes with wash buffer. Repeat twice.
8. Prepare working solution right before next step by adding one drop (20uL) of AEC chromogen to 1mL AEC substrate.
9. Add AEC working solution to the section and incubate for 20 minutes or until desired color occurs.
10. Wash slides for 3 minutes with wash buffer. Repeat once.
11. Rinse with distilled water and drain.
12. Add an adequate amount of the Mayer’s hematoxylin stain to the slide to cover the entire tissue surface.
13. Incubate for at least 5 minutes at room temperature.
14. Drain off the hematoxylin and wash the slide several times with distilled water.
15. Wash slide in wash buffer for 1 minute.
16. Wash the slide with distilled water.
17. Mount the slide. Add 1 -2 drops of mounting media to the center of the section. Carefully lay the cover slips on one edge and slide down the cover slips.

Section 4

1. Make sure the tissue surface stays **WET during the whole procedure.**
2. The AEC should generate a red color. Take pictures within 48 hours after the staining process is completed to prevent color from fading.
3. When working with AEC, use aqueous mounting media and hematoxylin that does not contain alcohol. AEC will dissolve in alcohol.
