# Supplementary figures and images for: Real-time PCR and immunohistochemistry detection of Wolbachia in adult Dirofilaria immitis from dogs treated with doxycycline and ivermectin
Source: Parasit Vectors. 2025 Feb 26;18:78. doi: 10.1186/s13071-025-06720-3 (PMC11866827; doi:10.1186/s13071-025-06720-3)

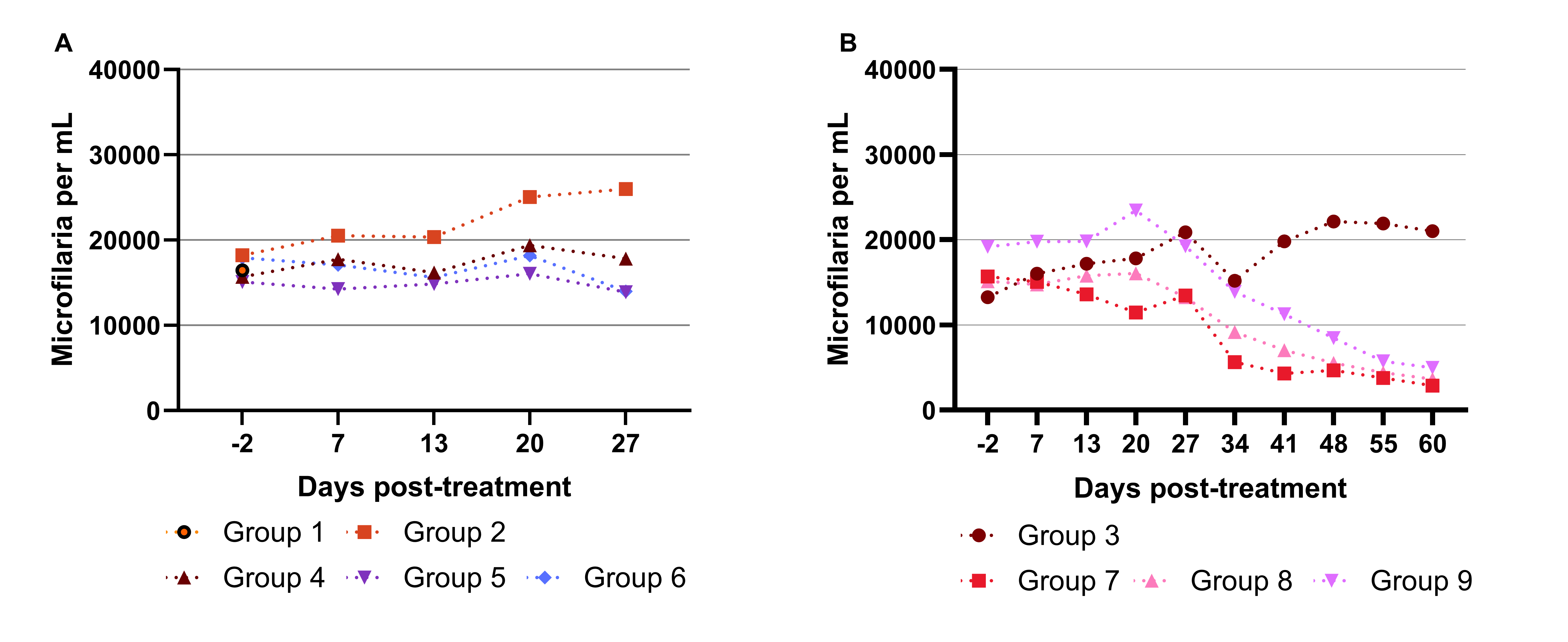

Supplement: Supplementary file 3 — Additional file 3. Supplementary Fig. S1. Mean microfilaria counts for groups. Fig. S1. Mean microfilaria counts for groups necropsied on days 0 and 30 (S1-A) and on day 60 (S1-B), beginning 2 days prior to the start of DOXY and IVM treatment. Groups 1–3: control groups with no treatment; groups 4, 7: 5 mg/kg DOXY; groups 5, 8: 7.5 mg/kg DOXY; groups 6, 9: 10 mg/kg DOXY. Doxycycline was given twice daily for 28 days. All treatment groups were given monthly IVM. [file 13071_2025_6720_MOESM3_ESM.tif]
